# Supplementary figures and images for: Enhanced transcriptional heterogeneity mediated by NF-κB super-enhancers
Source: PLoS Genet. 2022 Jun 1;18(6):e1010235. doi: 10.1371/journal.pgen.1010235 (PMC9191726; doi:10.1371/journal.pgen.1010235)

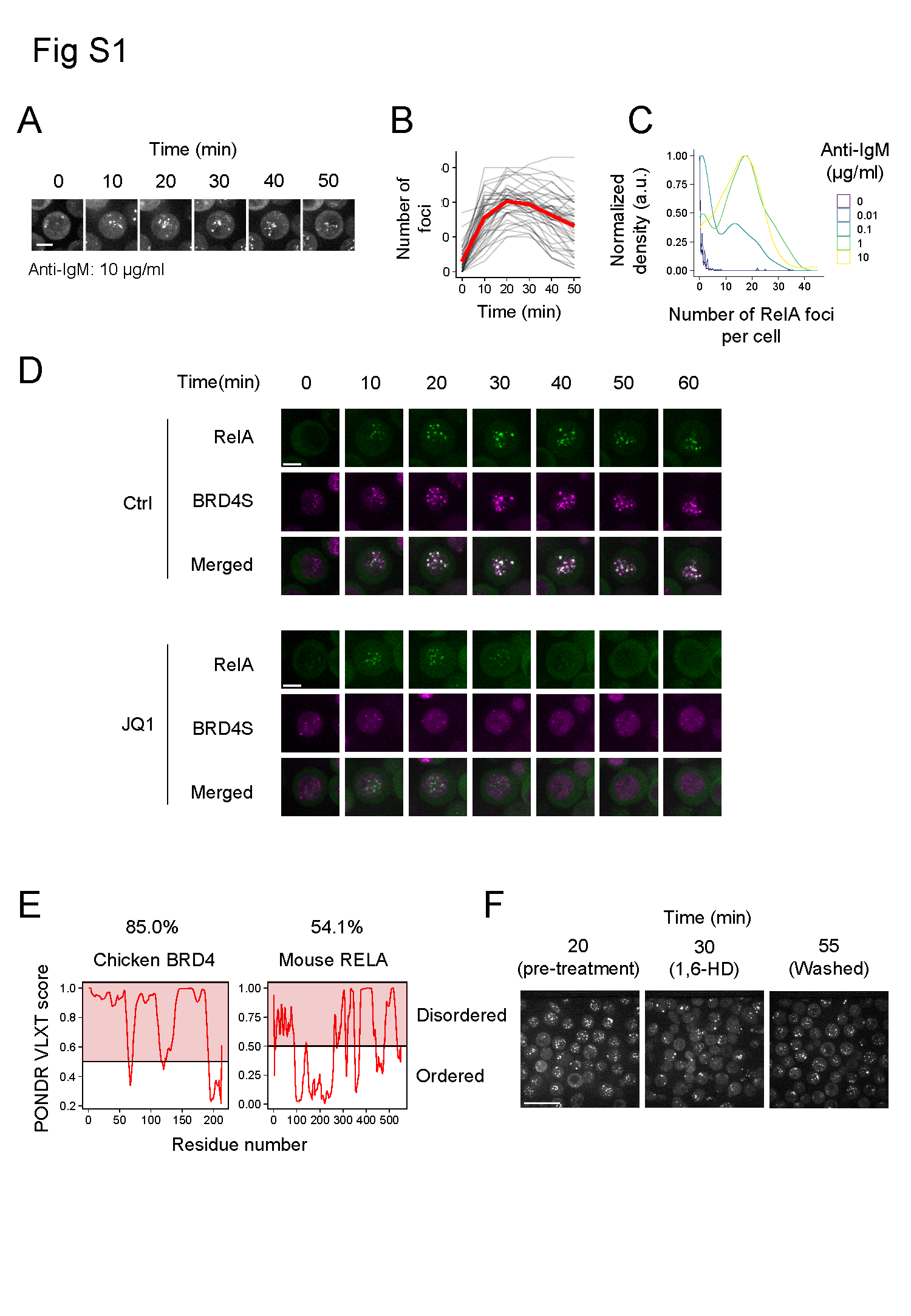

Supplement: S1 Fig — (A) Representative fluorescence micrographs of a single cell upon stimulation with 10 μg/ml anti-IgM (scale bar, 10 μm). (B) Changes in the number of foci detected in single-cells across time upon addition of 10 μg/ml anti-IgM (n = 49). The red line indicates the mean. (C) Distribution of RelA foci per cell upon stimulation with various doses of anti-IgM for 20 min. (D) Time-lapse fluorescence micrographs of DT40 cells co-expressing mKate2-BRD4S and RelA-GFP upon stimulation with 10 μg/ml anti-IgM and pre-treatment with JQ1 (5 μM) for 60 min (scale bar, 5 μm). (E) PONDR VLXT disorder scores of BRD4 and RelA. PONDR score more than 0.5: BRD4, 85.0%; RelA, 54.1%. (F) Representative fluorescence micrographs of a cell population stimulated with 10 μg/ml anti-IgM before treatment, after 1,6-hexanediol treatment, and upon washing (scale bar, 25 μm). (TIF) [file pgen.1010235.s001.tif]

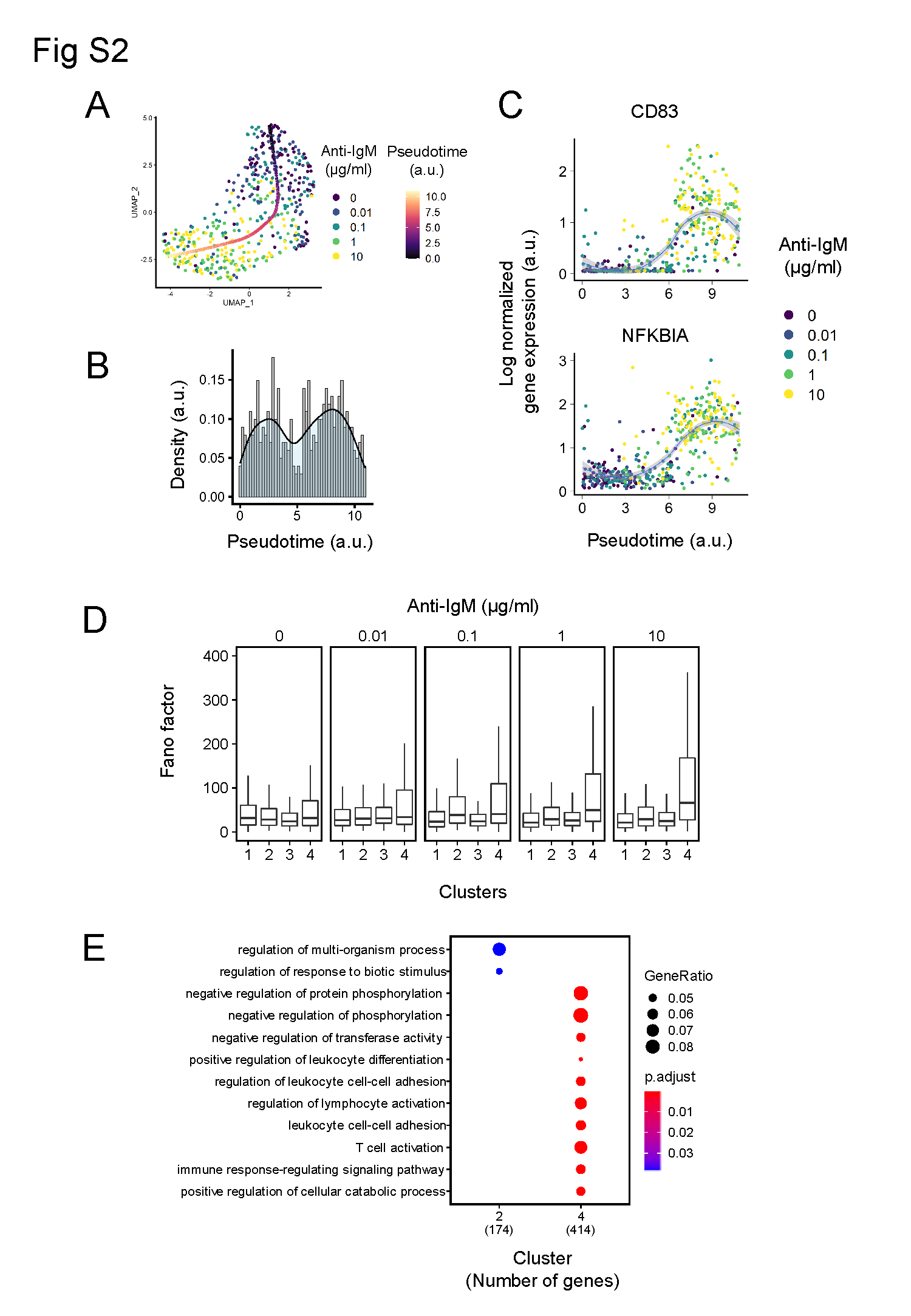

Supplement: S2 Fig — (A) Pseudo-time axis taken using a principal curve over a UMAP projection showing the doses of anti-IgM. (B) Distribution of cells across pseudo-time. (C) Gene expression of CD83 and NFKBIA in single-cells across pseudo-time. (D) Boxplot showing Fano factor of each heterogeneity cluster across different anti-IgM concentrations. (E) Biological processes gene ontology enrichment analysis for DEGs clustered according to Fano factor changes across anti-IgM doses. (TIF) [file pgen.1010235.s002.tif]

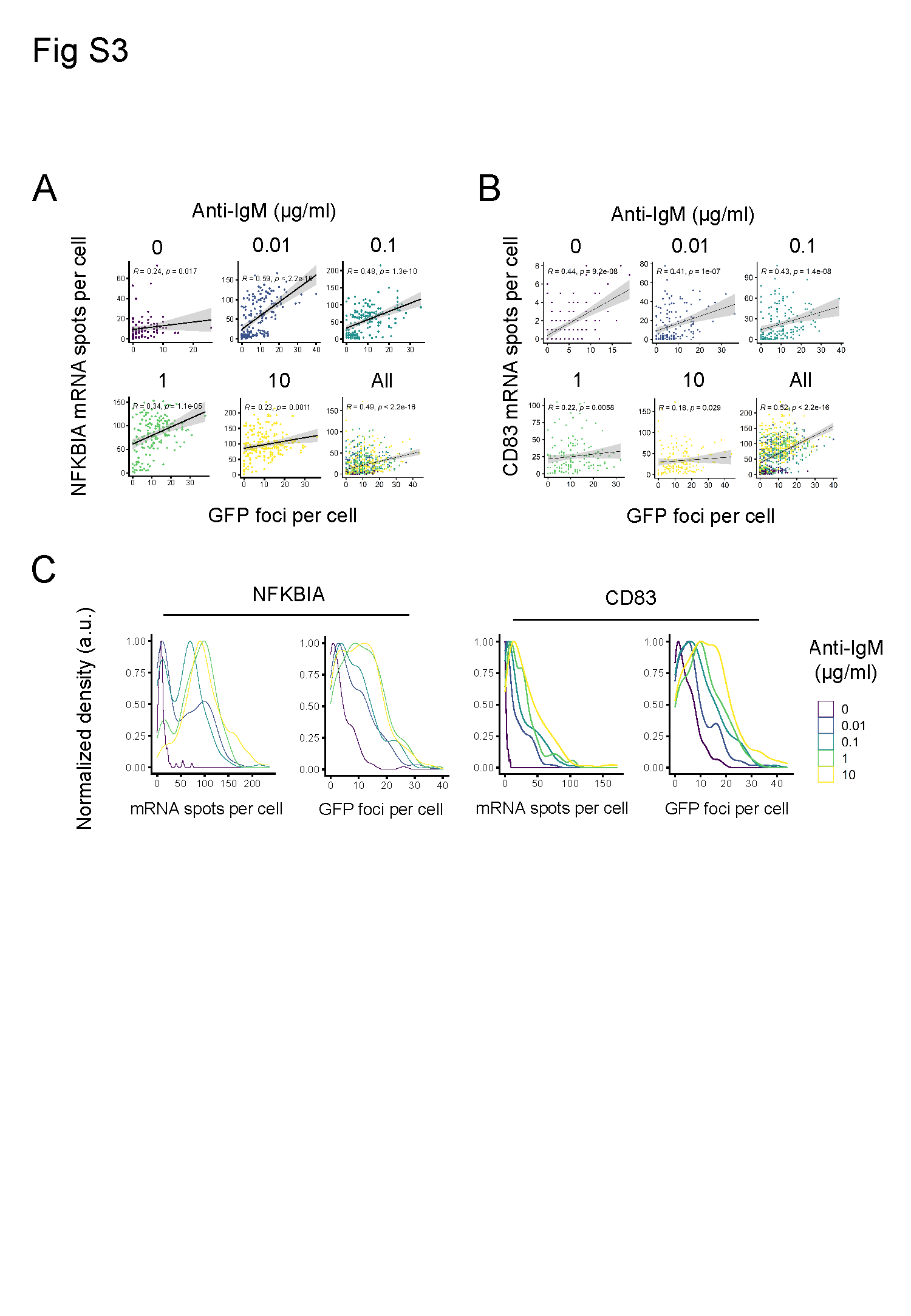

Supplement: S3 Fig — (A–B) Correlation plot between GFP foci and mRNA spots per cell for various doses of anti-IgM. (C) Density plot of RNA spots and GFP foci at various anti-IgM doses from the smRNA-FISH analysis. (TIF) [file pgen.1010235.s003.tif]

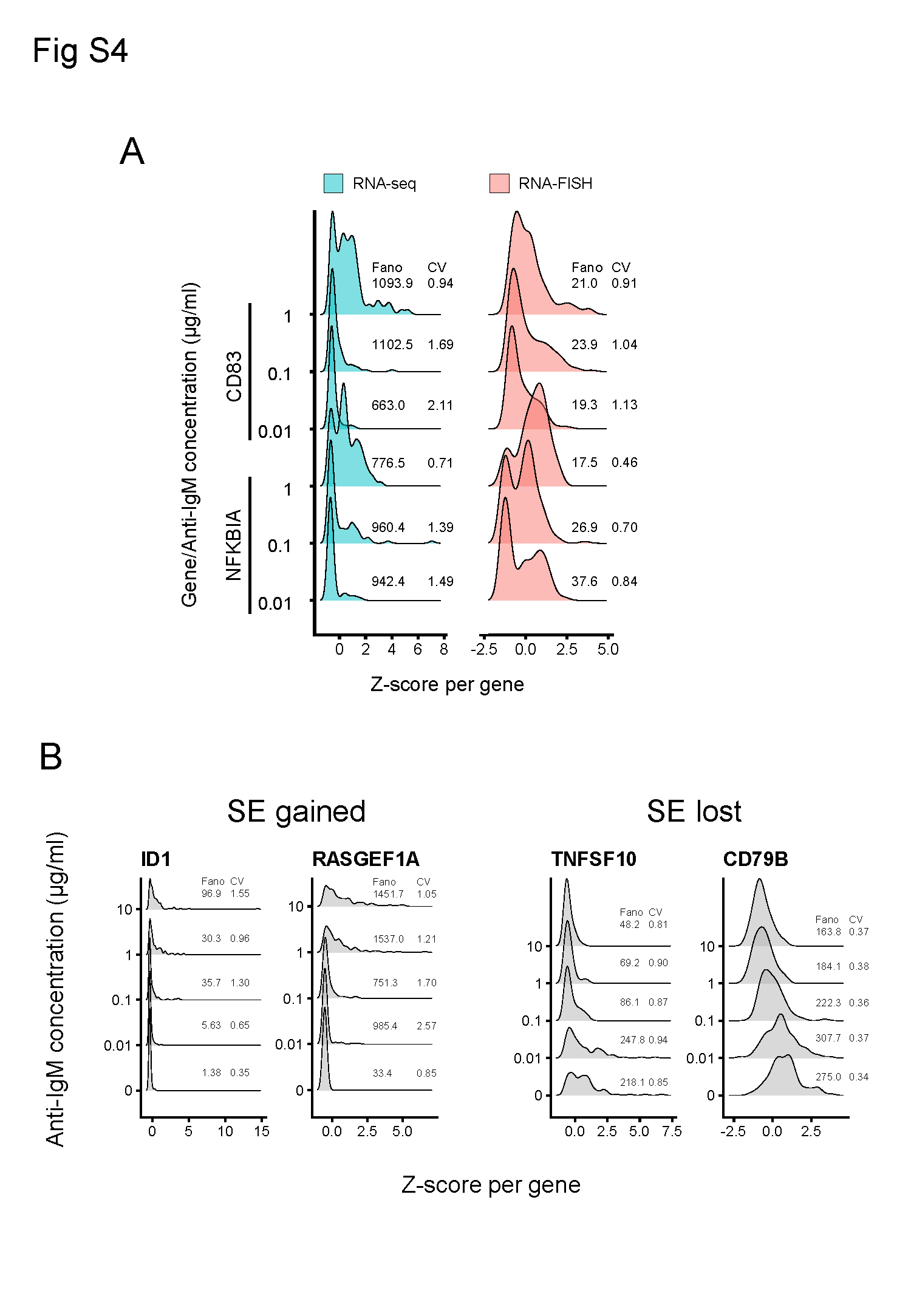

Supplement: S4 Fig — (A) Single-cell expression of CD83 (B cell activation marker) and NFKBIA (NF-κB target gene) obtained from scRNA-seq and smRNA-FISH at various doses of anti-IgM. (B) Single-cell expression of SE-annotated representative genes across anti-IgM doses. (TIF) [file pgen.1010235.s004.tif]

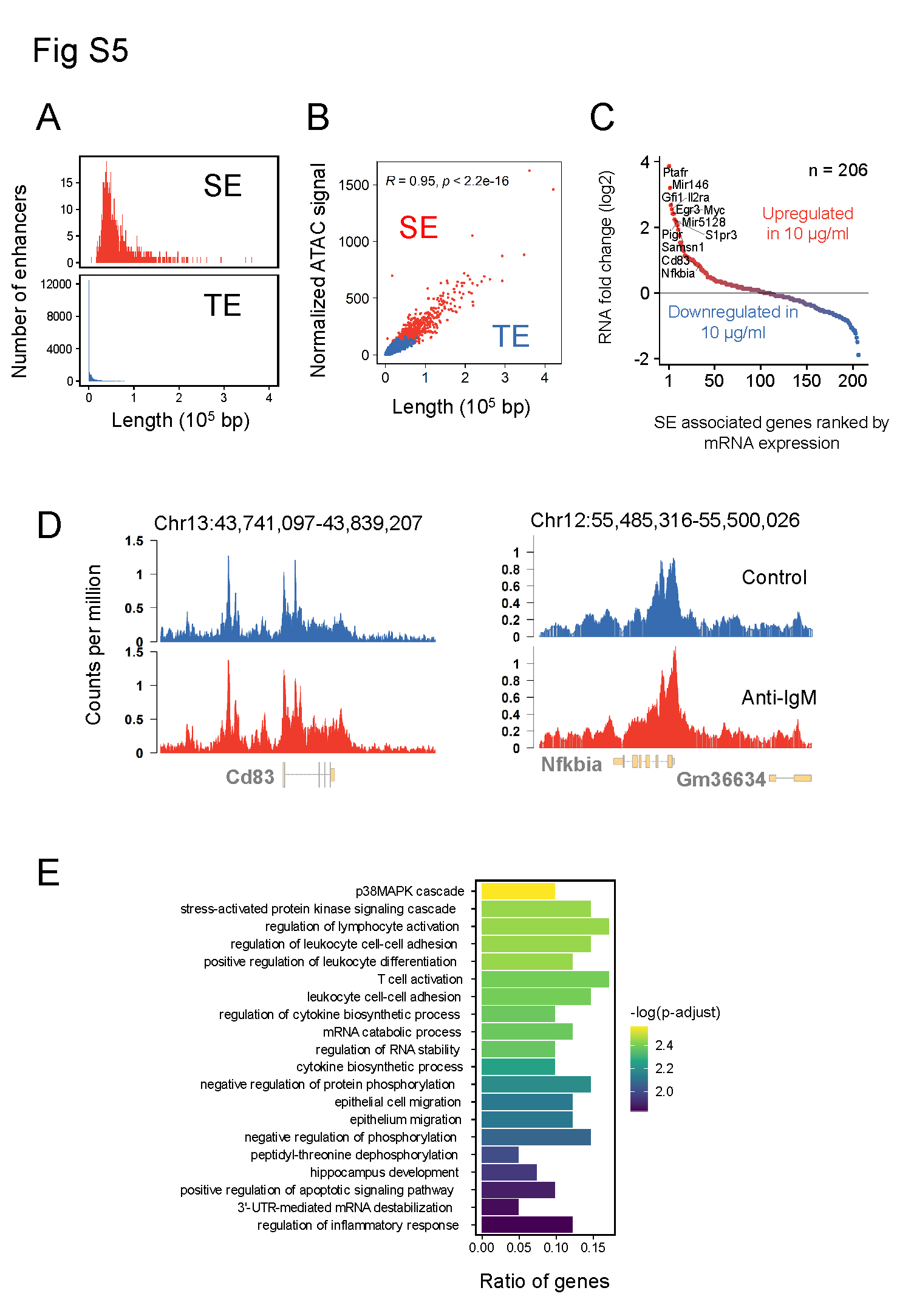

Supplement: S5 Fig — (A) Length of classified SE and TE in DT40 chicken B cells. (B) Correlation plot between enhancer length and normalized ATAC signal in DT40 chicken B cells. R = Pearson correlation coefficient. (C) Scatter plot of the mean fold-change of SE-associated genes between 10 μg/ml anti-IgM stimulated and control mouse primary B cells [8]. Note that SE was identified from H3K27Ac ChIP-seq data (D) Track view of Cd83 and Nfkbia ATAC-seq data of mouse primary B cells with 0 and 10 μg/ml anti-IgM [8]. (E) Biological processes gene ontology (GO) enrichment analysis of 52 genes with both gained SE (upper quantile) and upregulated RNA (upper quantile). (TIF) [file pgen.1010235.s005.tif]

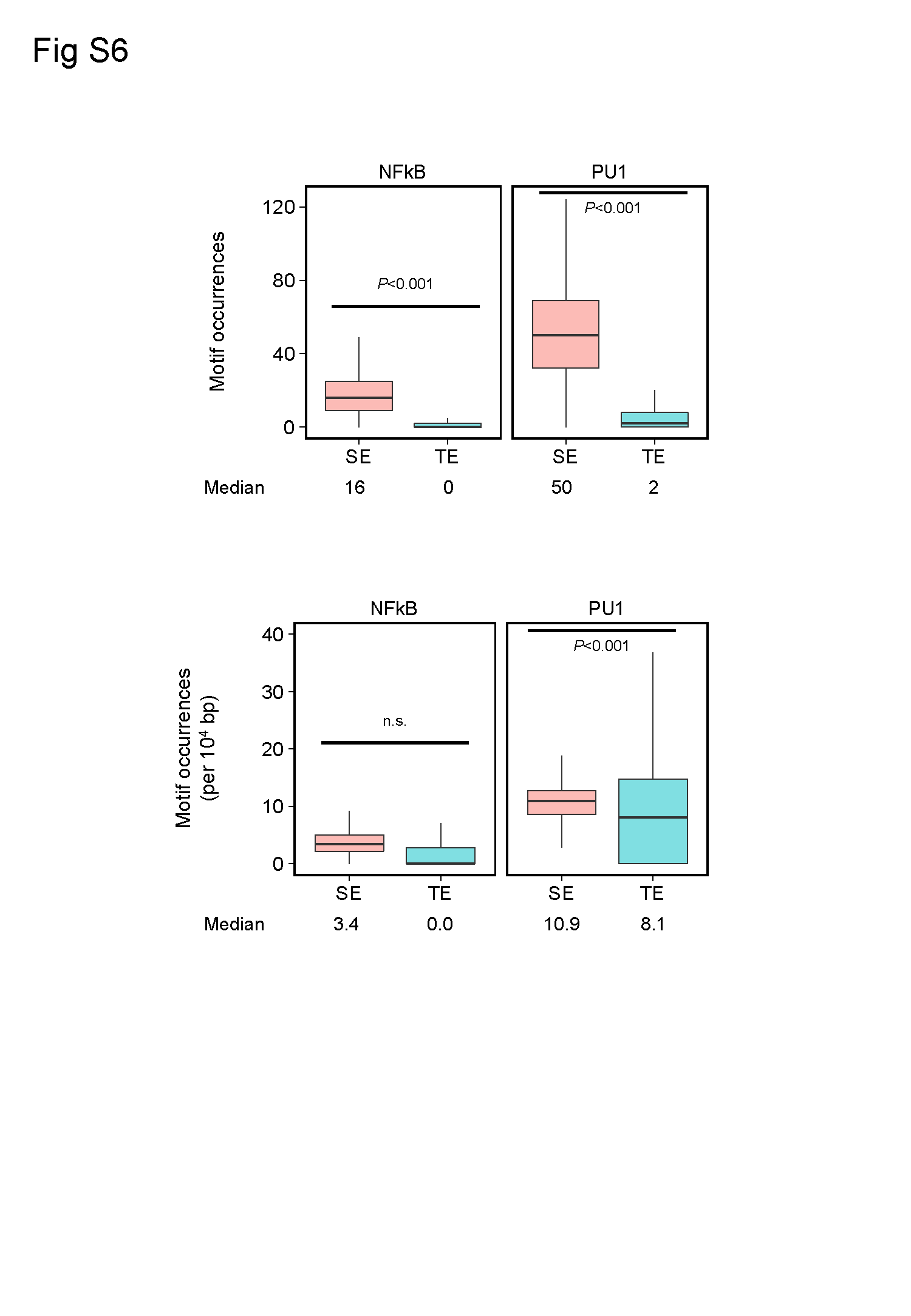

Supplement: S6 Fig — Motif occurrences of both NF-kB and PU.1 at TE (37,686 peaks) and SE (1,118 peaks) calculated using the “findMotifsGenome.pl” program with the “-find” option of Homer. The P-values were calculated using Welch’s t-test after undersampling (n = 280), n.s.: not significant. (TIF) [file pgen.1010235.s006.tif]

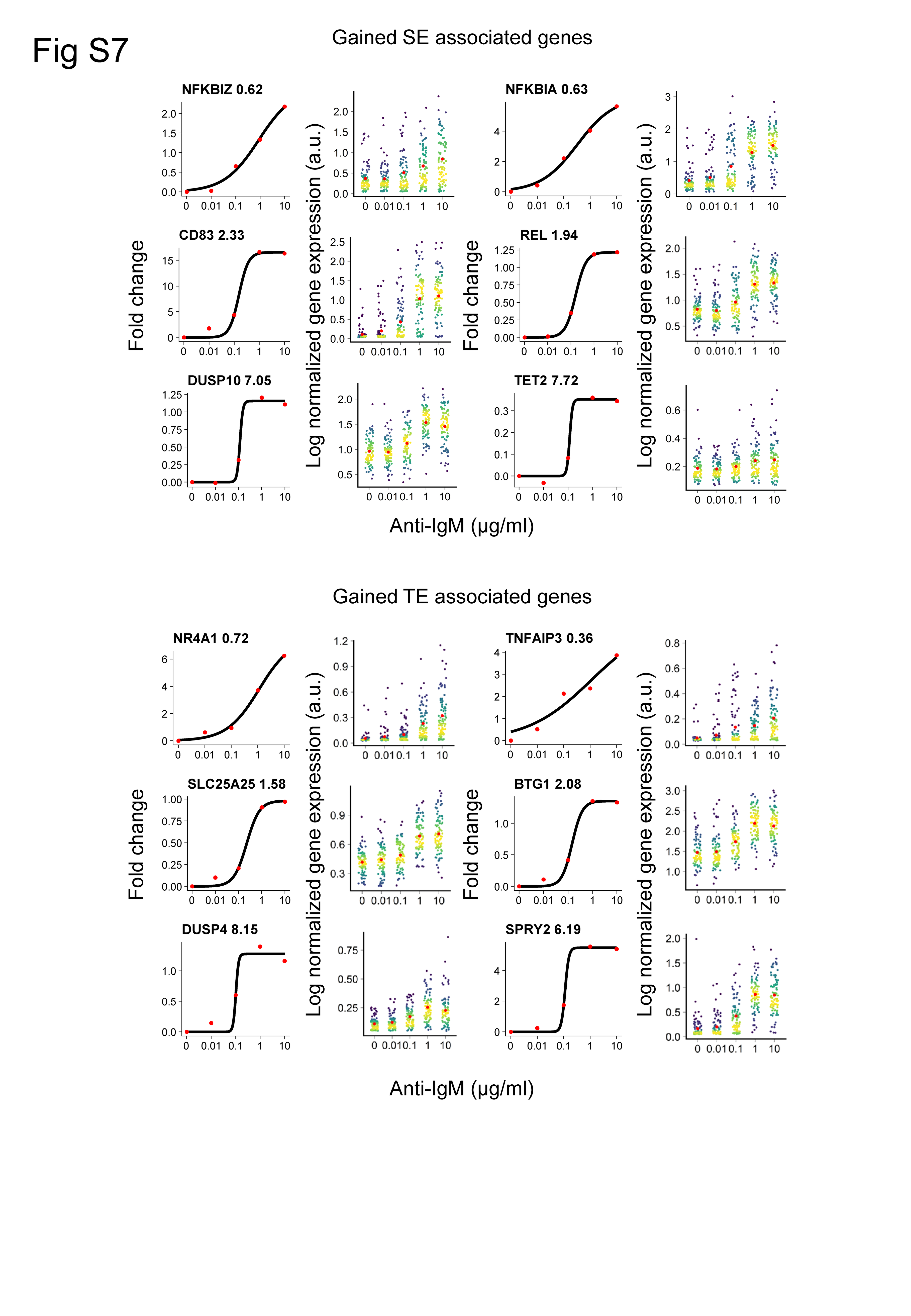

Supplement: S7 Fig — Hill function was fitted to the fold-change of mean gene expression compared to dose 0. Optimization was performed using the “optimx()” function of the R package optim, selecting the best optimization algorithm by the smallest residual sum of squares. Hill coefficient is shown beside the gene name. The scatter plot of normalized gene expression is shown on the right side. Red denotes mean. (TIF) [file pgen.1010235.s007.tif]

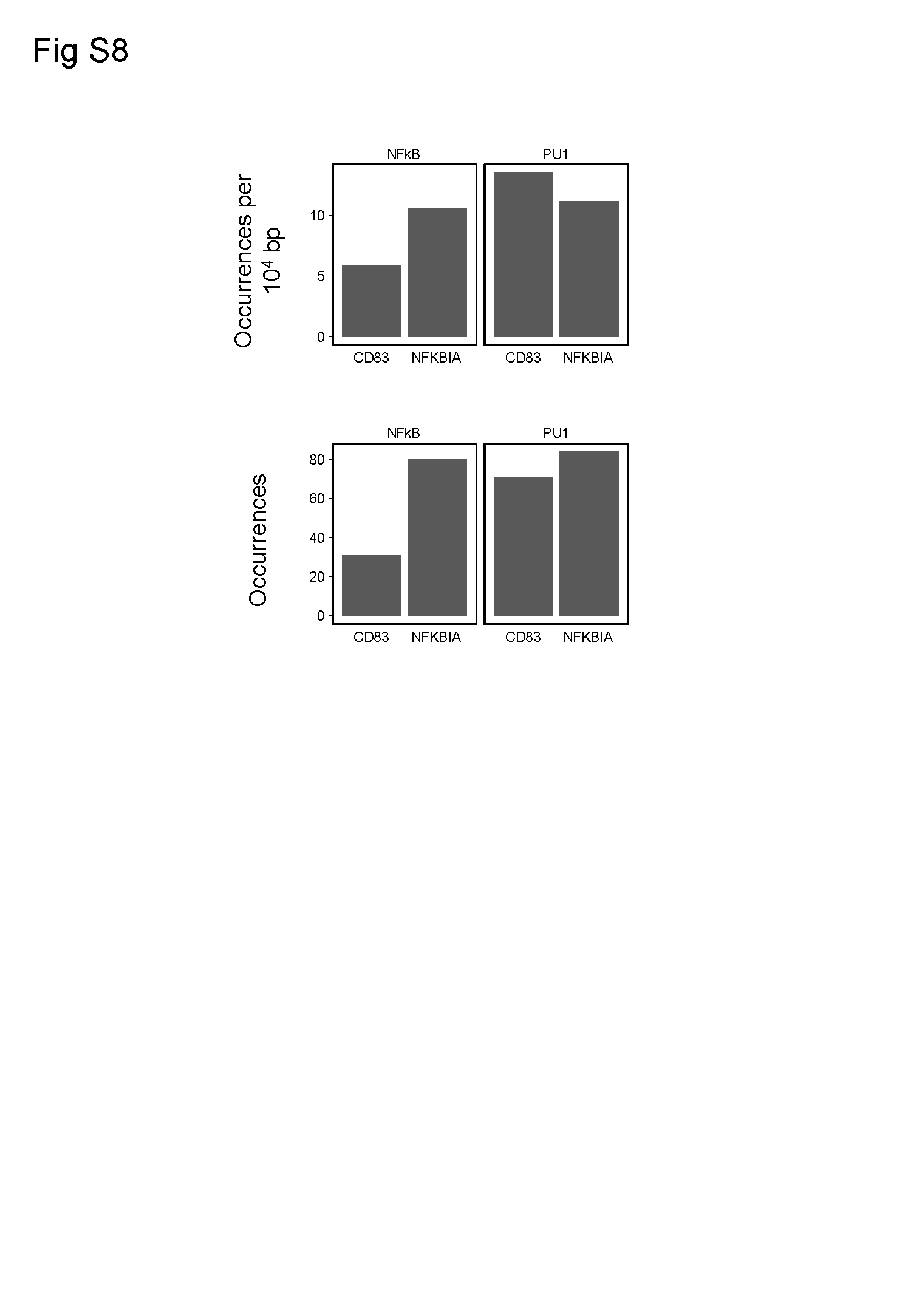

Supplement: S8 Fig — The motif number of PU.1 and NF-kB at merged SE regions for NFKBIA and CD83 calculated using Homer (See Methods). (TIF) [file pgen.1010235.s008.tif]

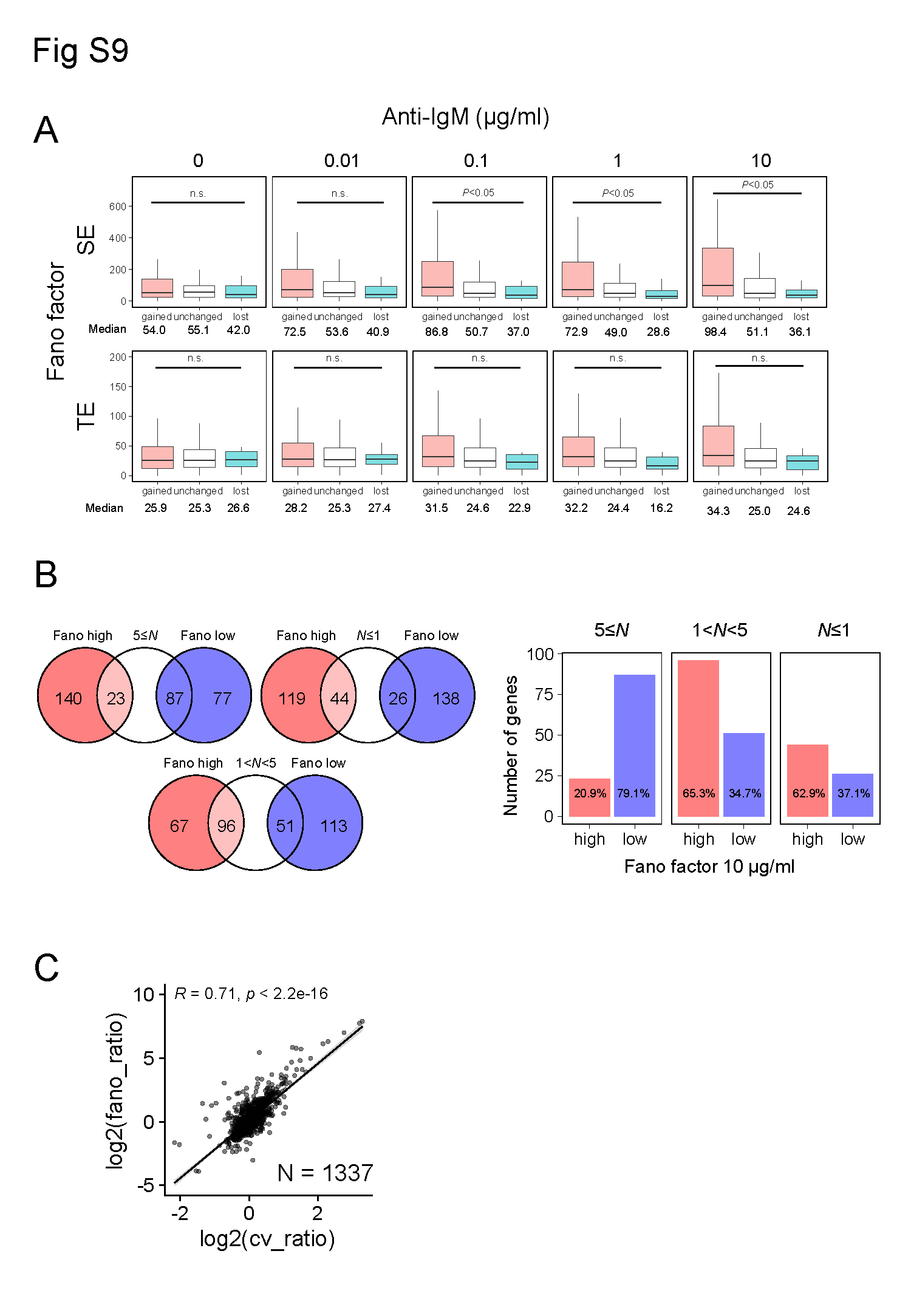

Supplement: S9 Fig — (A) Boxplot of Fano factor at all dose points for DEGs associated with TE and SE. Number of genes: Gained SE, 82; Unchanged SE, 100; Lost SE, 42; Gained TE, 242; Unchanged TE, 384; Lost TE, 21. P-value was calculated using one-way ANOVA with undersampling (n = 21), n.s.: not significant. (B) Venn diagram and bar plot of the number of genes with assigned Hill coefficient (N) below 1, above 5 and in between and subsequent Fano factor (10 μg/ml) below (low) and above the median (high). (C) Correlation plot of Fano factor and CV ratio between 10 ug/ml and 0 ug/ml anti-IgM stimulation. Correlation coefficients were calculated using Spearman’s rank correlation test. (TIF) [file pgen.1010235.s009.tif]

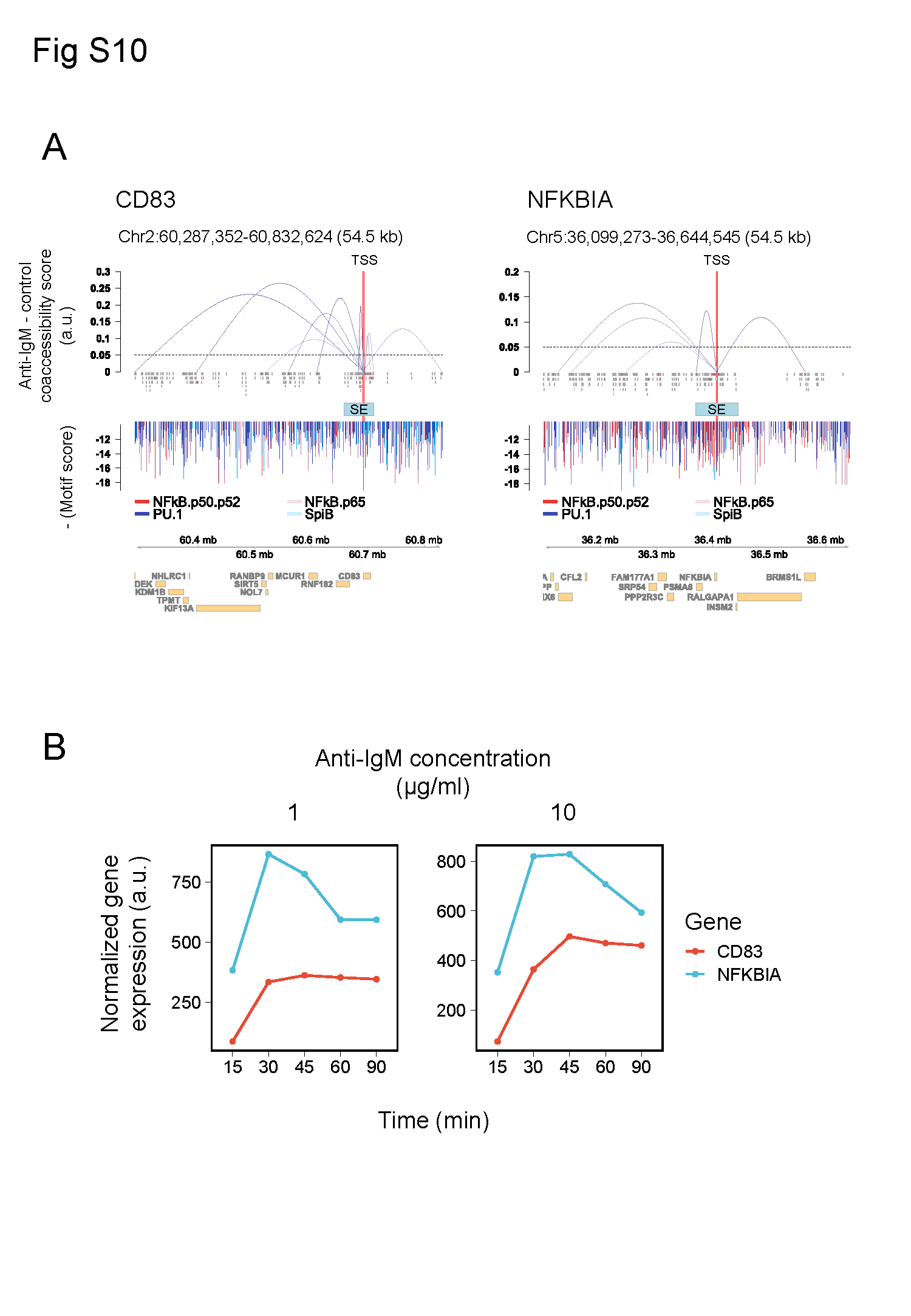

Supplement: S10 Fig — (A) Track view of NFKBIA and CD83 co-accessibility ± 1kb around the annotated transcription start site of and regions outside of the SE. (B) Time-course normalized gene expression of CD83 and NFKBIA obtained from microarray [42]. (TIF) [file pgen.1010235.s010.tif]

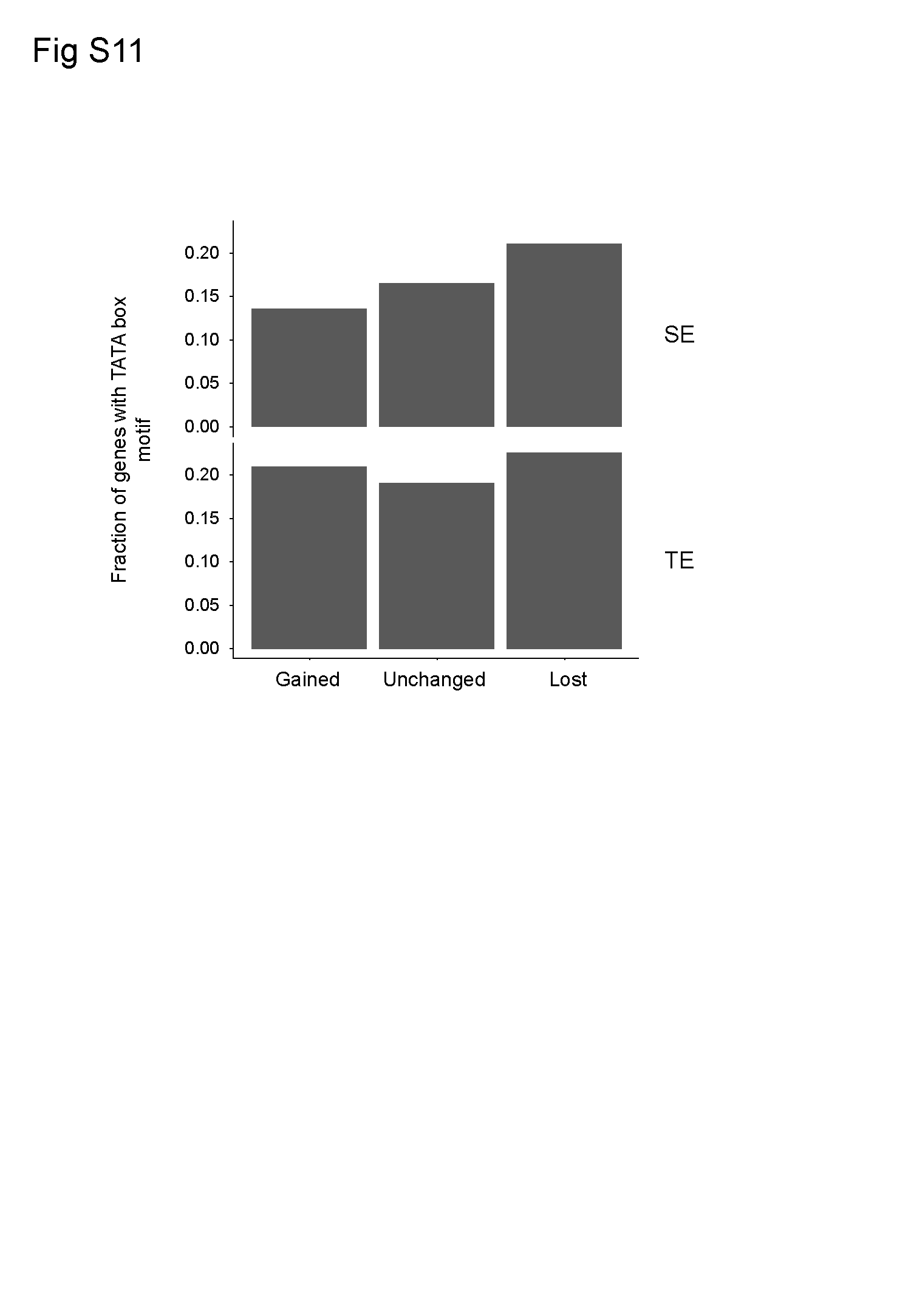

Supplement: S11 Fig — Motif number of TATA box at the promoters of TE and SE associated genes calculated using Homer (See Methods). (TIF) [file pgen.1010235.s011.tif]

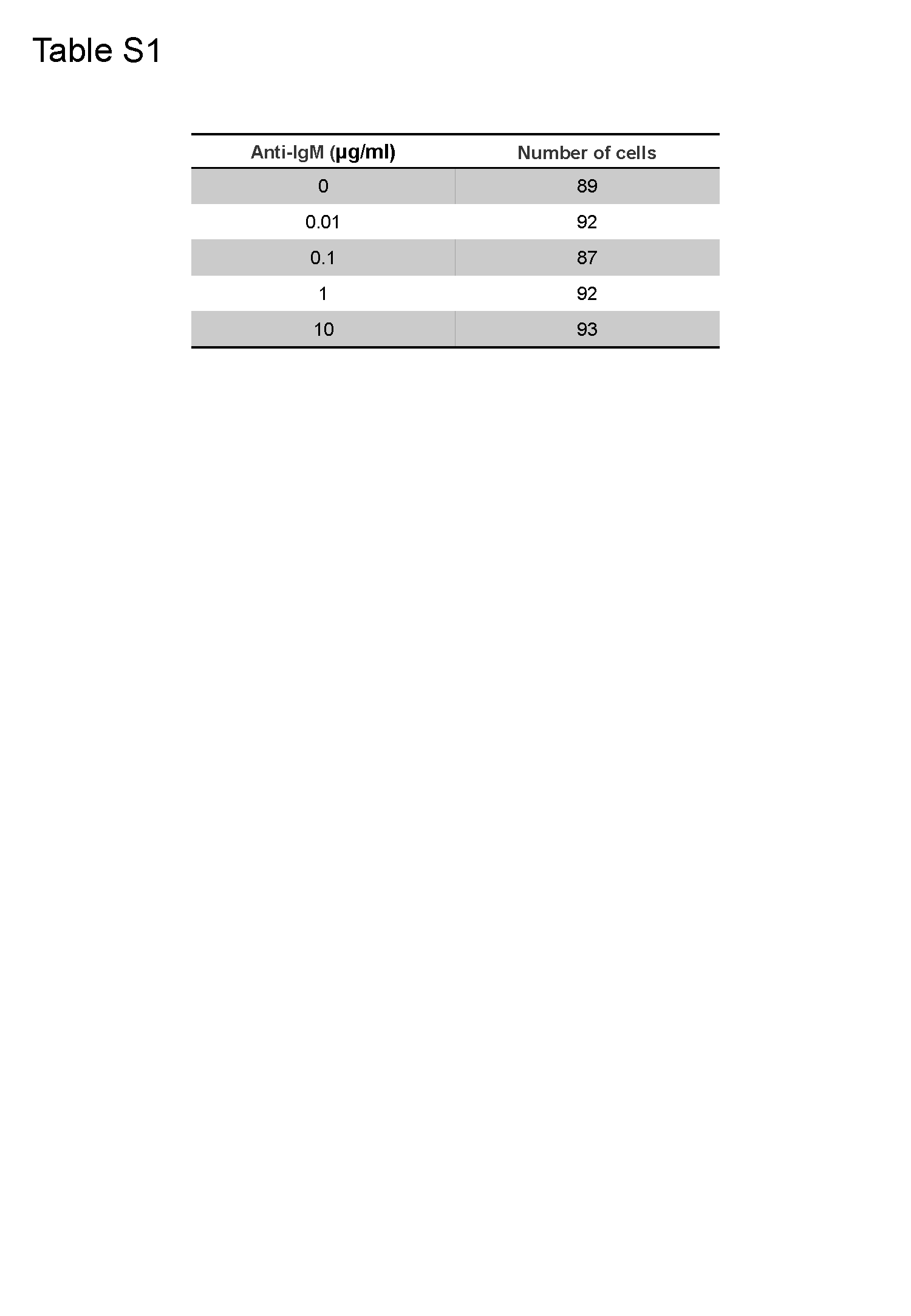

Supplement: S1 Table — (TIF) [file pgen.1010235.s012.tif]

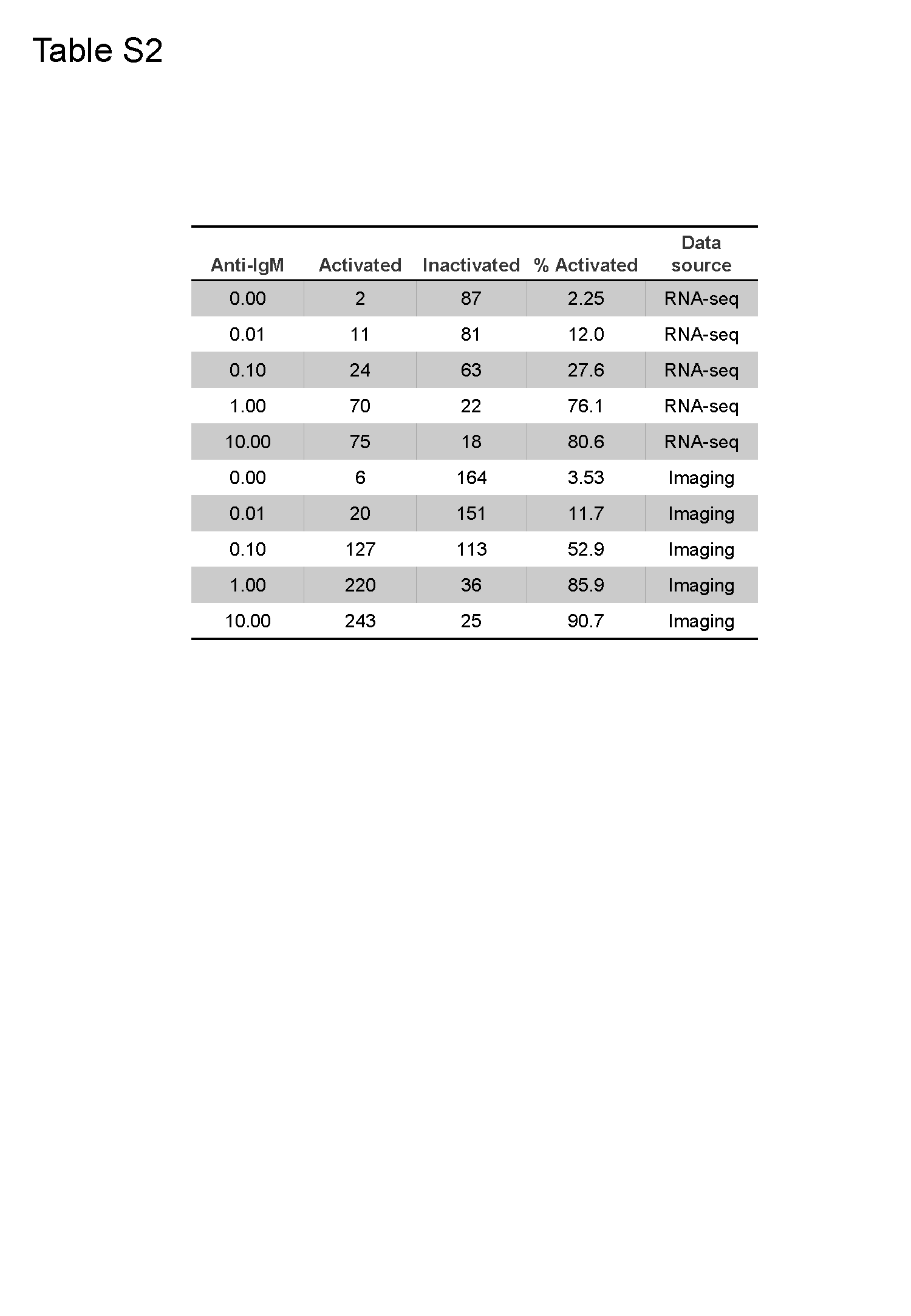

Supplement: S2 Table — (TIF) [file pgen.1010235.s013.tif]
